# Upper eyelid reconstruction: a short report of an eyelid defect following a thermal burn

**DOI:** 10.1186/1746-160X-5-26

**Published:** 2009-11-25

**Authors:** Francesco Inchingolo, Marco Tatullo, Fabio M Abenavoli, Massimo Marrelli, Alessio D Inchingolo, Roberto Corelli, Angelo M Inchingolo, Gianna Dipalma

**Affiliations:** 1 Department of Dental Sciences and Surgery, General Hospital, Bari, Italy; 2 Department of Otorinolaringoiatry, Hospital "Fatebenefratelli", Rome, Italy; 3 Department of Maxillofacial Surgery, Calabrodental, Crotone, Italy

## Abstract

While the principles of eyelid reconstruction are well-established, achieving good functional and aesthetic reconstruction remains challenging.

This communication presents a technique that we used on a young patient with an eyelid defect following a thermal burn. The patient was operated on to reconstruct the entire upper eyelid using, as a posterior lamella, a mucochondrial autologous graft taken from the ala of the nose as a tarsus and conjunctiva substitutes that were sutured to the Elevator palpebrae superioris aponeurosis and muscle. On the other hand, to reconstruct the anterior lamella, which consists of skin and muscle, the surgeons used a myocutaneous temporal flap taken from the region immediately lateral to the external canthus of the palpebral region, and which, after being isolated following a drawing of the upper eyelid to be reconstructed, was rotated and then sutured to the posterior lamella using the orbicularis oculi muscle as a pedicle.

## Introduction

While the principles of eyelid reconstruction are well-established, achieving good functional and aesthetic reconstruction remains challenging (1).

A variety of techniques are now available, depending on the type of lesion and its cause. This communication presents a technique that we used on a young patient with an eyelid defect following a thermal burn, which we recently encountered.

## Case Report

**Figure 1:**
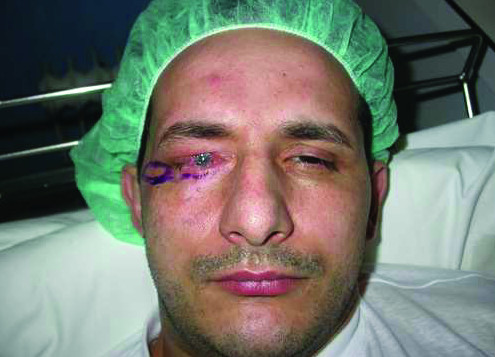
**Drawing of the orbicularis oculi myocutaneous flap and surgical incisions**.

**Figure 2:**
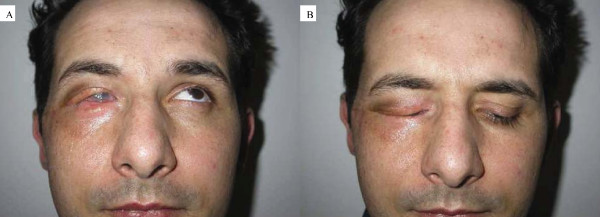
**Postoperative result two months later, with palpebra that can move up (2a) and down (2b)**.

The 37 year-old patient from a small town in northern Italy, was welding when he was hit by a spray of liquid metal on the periorbital region that burned the upper eyelid and part of the lower eyelid. The patient was treated by eye-specialists at the local hospital with multiple debridements of the palpebral lesions, that take several weeks to heal, save for the complete loss of the upper eyelid, albeit partially maintaining the medial and lateral canthus, along with a loss of the ciliary margin of the entire lower eyelid. In the weeks which followed, the inflammation of the eyeball, which was undamaged by the metal but irritated due to a lack of palpebral protection, worsened further, partially compromising the eye's functionality. So, surgery was scheduled to reconstruct the eyelid in a single operation, because of the distance the patient had to travel, which would also provide satisfactory functional and aesthetic results. The patient was operated on to reconstruct the entire upper eyelid using, as a posterior lamella, a mucochondrial autologous graft taken from the ala of the nose as a tarsus and conjunctiva substitutes that were sutured to the levator palpebrae

superioris aponeurosis and muscle. On the other hand, to reconstruct the anterior lamella, which consists of skin and muscle, the surgeons used a myocutaneous temporal flap taken from the region immediately lateral to the external canthus of the palpebral region, and which, after being isolated following a drawing of the upper eyelid to be reconstructed (Fig. 1), was rotated and then sutured to the posterior lamella using the orbicularis oculi muscle as a pedicle. The donor area was closed, following the ample undermining of the edges, with a linear scar (hardly noticeable) on the temporal area. The incision performed on the lower palpebral rim, necessary for the preparation of the orbicularis muscle as a myocutaneous temporal flap, was used to place a composite graft taken from the right auricular concha in order to lift and provide greater support for the lower eyelid. The patient's postoperative recovery was normal, and within 7 days he was able to return home, to be cared for once more by local eye-specialists in continuing his medical treatment of the eyeball. When the patient returned two months later for a check-up, the aesthetic and functional result appeared satisfactory. In fact, the new upper eyelid with a stable lid margin could open and close properly in spite of the ongoing presence of residual edema (Fig. 2 a-b).

## Discussion

We, thus, consider the possible use of the myocutaneous temporal flap as a substitute for the anterior lamella of the upper eyelid in the reconstruction of the complete eyelid to be extremely satisfactory. Thanks to the fact that the orbicularis oculi muscle beyond the orbital margins covers the surface of the frontalis, temporalis, and other muscles, this pedicle, previously mentioned by Yoshimura et al. in 1991 (2), provides the possibility of further transferring orbicularis muscle to the missing area when it is not possible to use the lower eyelid as a donor area, with skin whose characteristics are similar to those of the upper eyelid and with absolutely sure vascularization since it contains branches of the angular artery and the infraorbital artery in its pedicle. Making use of a muco-cartilaginous graft taken from the ala of the nose is also advantageous, since it reproduces the firmness and features of the posterior lamella formed by the tarsal plate and the conjunctiva, without altering either the functionality or the aesthetics of the ala of the nose.

## Conclusion

We, therefore, believe our experiences, which can easily be reproduced in cases of total or subtotal full-thickness defects of the upper eyelid, will be of interest.

## Consent

Written informed consent was obtained from the patient for publication of this case report and accompanying images. A copy of the written consent is available for review by the Editor-in-Chief of this journal.

## Competing interests

The authors declare that they have no competing interests.

## Authors' contributions

FI: conceived of the study and participated in its design and coordination; MT: drafted the manuscript and participated in the design of the study; FMA: conceived of the study and participated in its design and execution; MM: participated in the design of the study; ADI: revised the literature sources; RC: participated in design and execution of the study; AMI: documented surgery with digital pictures; GD: participated in the design of the study; All authors read and approved the final manuscript.
